# Vision-Based Tactile Sensor Mechanism for the Estimation of Contact Position and Force Distribution Using Deep Learning

**DOI:** 10.3390/s21051920

**Published:** 2021-03-09

**Authors:** Vijay Kakani, Xuenan Cui, Mingjie Ma, Hakil Kim

**Affiliations:** 1 Information and Communication Engineering, Inha University, 100 Inharo, Nam-gu, Incheon 22212, Korea; vjkakani@inha.ac.kr (V.K.); xncui@inha.ac.kr (X.C.); 2 VisionIn Inc. Global R&D Center, 704 Ace Gasan Tower, 121 Digital-ro, Geumcheon-gu, Seoul 08505, Korea; mjma@vision-in.co.kr

**Keywords:** vision-based tactile sensor, deep learning, contact position, contact area, force distribution

## Abstract

This work describes the development of a vision-based tactile sensor system that utilizes the image-based information of the tactile sensor in conjunction with input loads at various motions to train the neural network for the estimation of tactile contact position, area, and force distribution. The current study also addresses pragmatic aspects, such as choice of the thickness and materials for the tactile fingertips and surface tendency, etc. The overall vision-based tactile sensor equipment interacts with an actuating motion controller, force gauge, and control PC (personal computer) with a LabVIEW software on it. The image acquisition was carried out using a compact stereo camera setup mounted inside the elastic body to observe and measure the amount of deformation by the motion and input load. The vision-based tactile sensor test bench was employed to collect the output contact position, angle, and force distribution caused by various randomly considered input loads for motion in *X*, *Y*, *Z* directions and $R_{x}R_{y}$ rotational motion. The retrieved image information, contact position, area, and force distribution from different input loads with specified 3D position and angle are utilized for deep learning. A convolutional neural network VGG-16 classification modelhas been modified to a regression network model and transfer learning was applied to suit the regression task of estimating contact position and force distribution. Several experiments were carried out using thick and thin sized tactile sensors with various shapes, such as circle, square, hexagon, for better validation of the predicted contact position, contact area, and force distribution.

## 1. Introduction

Vision-based processing has been a part of inference in many interdisciplinary fields of research [1,2,3]. The usage of vision-based tactile sensors in industrial applications has grown over the past two decades with the rise in the standard of imaging sensors [4,5,6]. Usually, the tactile sensors can perceive the physical aspects of any object, which indeed guides the handling of the object in terms of strength applied to interact with them [7]. On the contrary, visual sensors, such as cameras, do not interact with the objects physically. Instead, they retrieve the visual cues from the imaging patterns of the objects in various modes [8]. The perceiving capability can be improved by using information, such as visual patterns, adapted force, and contact location, retrieved from the visual sensors without having to interact with the object in a physical manner [9]. This can be made possible using deep learning, which utilizes the data collected from vision sensors, along with the parameters, such as contact position, and force distribution, and trains on it to predict the output parameters in the future [10].

### 1.1. Background

**Figure 1:**
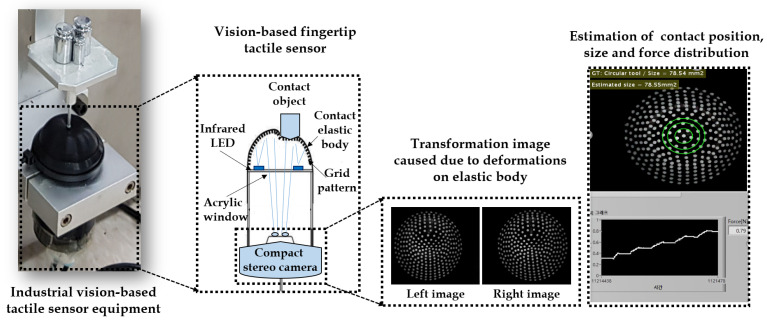
Principle of detection in vision-based tactile sensor technology.

The vision-based tactile sensing mechanism is developed using the same scheme, where the camera is mounted inside the elastic tactile sensing fingertip. Whenever the object is in touch with the fingertip, the camera gets the transformed grid pattern used to estimate the contact position and force distribution. The correlation between the input load force, contact position and transformed image captured by the camera sensor can be learned throughout various scenarios [11]. In this case, the vision-based tactile sensor technology gets rid of the need for the usage of separate traditional array type tactile sensor strips which are usually less durable and prone to large signal processing burden and breakage [12]. Furthermore, this type of visual-based tactile sensor is more like a single element type with no physical interaction with the elastic body. In the worst-case scenario, the elastic part can be replaced if damaged but the visual sensor always stays safe [13]. Additionally, indirect contact with the elastic body means the signal processing burden reduces by tenfold even if the detection area increases. The image acquisition process in the context of a visual-based tactile sensor can be observed in Figure 1. The industrial vision-based tactile sensor equipment used in this study is depicted, along with the transformed stereo image pair caused due to deformations on the elastic body.

### 1.2. Problem Statement

- The problem statement of this study is to predict the force distribution and contact position parameters that are to be estimated by the trained deep learning network using the training data acquired from the visual tactile sensor setup.

**Figure 2:**
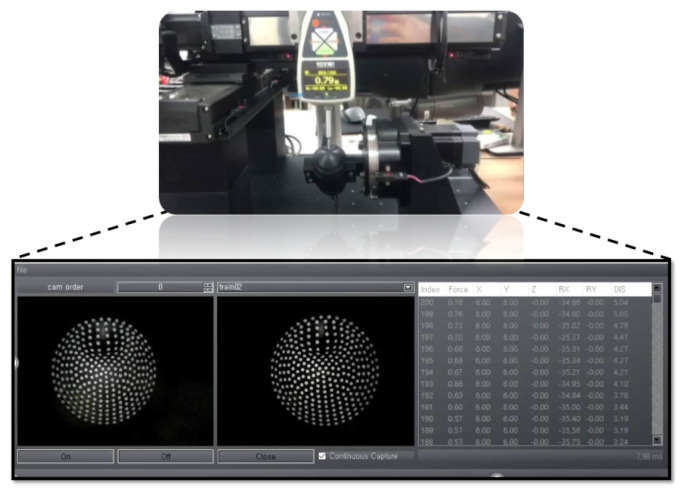
Problem statement of vision-based tactile sensor mechanism for the estimation of contact location and force distribution using deep learning: Data acquisition and training and inference stage.

Usually, the common inference problems that deep learning models are usually trained on are classification and detection problems which are straightforward using the class labels and corresponding training samples to predict/detect the target class objects. In this study, the models must be tailored to match the problem statement of estimating continuously varying quantities, such as contact location and force distribution. Therefore, the problem statement for this study focuses on implementing a customized problem-specific regression model through transfer learning on top of pre-trained deep learning network architecture. This means the training data has to be collected under diverse conditions, such as various inputs loads with different object shapes, tactile sensor thickness, etc. This collected data then has to be paired with the stereo camera samples (which captures the deformation of the elastic body) in terms of right and left images. This collective data has to be properly handled and pre-processed to train the regression network for better prediction of contact position and force distribution, as shown in Figure 2.

### 1.3. Purpose of Study

The primary purpose of this study is to develop a learned vision-based tactile sensor mechanism that uses indirect contact with the object to estimate the force and contact position of the impact when the object is interacting with the elastic body. In this study, the deep learning has been utilized as a tool for the training of tactile sensing mechanism w.r.t various parameters, such as images, input loads, contact positions, etc. As an underlying study, aspects, such as the development of the tactile fingertips and optimal setup of the compact stereo system, were detailed for practical purposes. Accordingly, issues, such as materials used in the manufacturing of tactile fingertips and their relative thickness, were discussed to enable the readers to understand the employed test bench equipment in detail. The usage of deep learning as a training and testing tool has been clearly described and the implementation details were explained to make a point regarding how to customize the domain-related network model into a problem-based use-case network model. In other words, this work focuses on detailing the transfer learning of domain-specific classification pre-trained network model, such as VGG16 [14], to deal with the regression problem of estimating the contact position and force distribution. In addition, this work illustrates the simple Yet, effective data pre-processing techniques that can enhance visual-tactile activity detection by a significant degree. The main contributions are as follows:

- employing deep learning for the transfer learning of VGG16 classification pre-trained network model; and
- validating the vision-based tactile sensor system to examine the estimation of contact position, contact area, and force distribution using thick and thin tactile sensors with various shapes.

The paper is organized as follows. Section 2 thoroughly discusses the previous works and their characteristics regarding the usage of computer vision/deep learning in vision-based tactile sensor technology. Section 3 explains the overall materials and methodologies utilized in this study. All the aspects, such as overall system installation, stereo camera setup, manufacturing, and practical issues, related to the tactile fingertips, deep learning network architecture, and transfer learning methodology are detailed in this section. Section 4 describes the tactile sensor experiments and related evaluation metrics. Section 5 reports the results and related discussions based on the applied deep learning methodology to estimate the tactile contact position and force distribution. Finally, Section 6 concludes the paper with a summary.

## 2. Literature Review

### 2.1. Vision-Based Tactile Sensor Technology

The practice of employing camera sensors to estimate the contact position and force distribution is actively researched in the past decade [15]. The vision sensors are compactly embedded in the tactile sensing mechanism such that the deformations in the elastic body is transformed as tactile force, contact position-based information [16]. With the increase in the pixel resolution of the visual sensors, the vision-based tactile sensitivity has also improved. Researchers have employed image processing and computer vision techniques to measure the force and displacement of markers [17]. The patterns on the deformed materials are analyzed using low-level image processing algorithms and support vector machines [18], and some studies even approached the problem of determining the contact force and tactile location in a machine learning perspective [19]. Some other studies adapted the usage of dynamic vision sensors and depth sensors for tactile sensing [20]. With the accessibility of compact circuit technologies and high spatial resolution vision systems, some studies were able to report 3D displacement in the tactile skins [21]. A few other works tried to embed multiple camera sensors inside the tactile sensor to retrieve the best possible internal tactile force fields [22]. On the other hand, there has been an appeal and enthusiasm towards the learning-based approaches inculcating deep learning for the estimation of tactile information [23]. The visual-based tactile sensing mechanism can be typically classified into two approaches, such as traditional image processing/computer vision-based methods and learning-based methods. In traditional image processing/computer vision methods, various low-level image manipulating techniques are employed to enhance the images retrieved from the deformation source [24]. Often, the traditional methods are directly working on the images retrieved from the input sensor. This enabled devising a pipeline that does not require any training data before the inference. On the contrary, the learning-based techniques heavily rely on the training data for the enhancement of the performance [25].

### 2.2. Previous Works

In the past decade, few studies were proposed in the context of using the vision-based technique in tactile sensing mechanism. Begej et al. [26] pioneered the usage of the vision-based tactile sensor for measuring the contact force and internal reflection. Lepora et al. [27] reported their studies on implementing super-resolution optical tactile sensor which can localize the contact location, as well as to measure the contact force. Ito et al. [28] proposed a method to estimate the slippage degree using a vision-based tactile mechanism with extensive experiments. Yang et al. [29] focused on analyzing the texture of the material using the micro RGB camera in the context of tactile finger instrumentation. A few studies, such as Corradi et al. [30] and Luo et al. [31], used the vision-based tactile mechanism to recognize various objects. There were also a few remarkable studies by Piacenza et al. [32] which accurately estimated the contact position with indentation depth prediction using visual-tactile sensors.

The work from Johnson et al. [33] demonstrating the measurement of surface texture and shape using their photometric stereo technology has gained prominence in the field. Later these studies were further modified to measure the normal and shear force and were reported in Johnson et al. [34] and Yuan et al. [35] The learning-based methods were employed by a few researchers, like Kroemer et al. [36] and Meier et al. [37], for the estimation of force exhibited in the tactile behavior. Especially, Meier et al. [37] used convolutional neural networks to detect the online slip and rotations. Similarly, Chuah et al. [38] used artificial neural networks (ANN) to improve the accuracy in estimation of normal and shear force. They employed an automatic data collection procedure to acquire the footpad while moving through various trajectories. The concept of transfer learning help speeds up the process of adapting learning-based mechanisms into the vision-based tactile sensing tasks. There are many studies, such as References [39,40,41], that adapted the transfer learning in the context of Convolutional Neural Networks (CNN) to attain better results in terms of determining the force and other tactile aspects. The details of the summarized vision-based tactile sensing techniques are stated in Table 1.

## 3. Materials and Methods

### 3.1. System Installation and Flow Schematic

**Figure 3:**
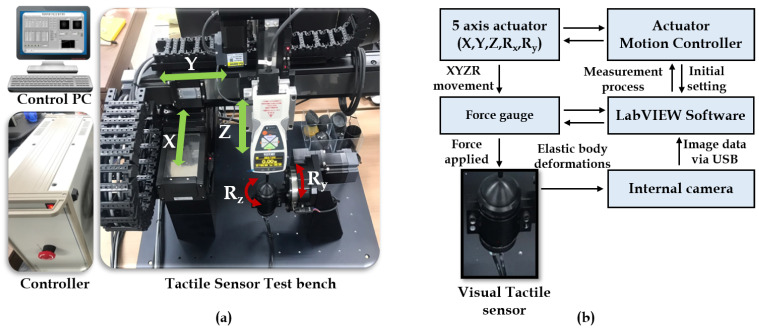
Equipment setup and schematic: (**a**) Overall system installation. (**b**) Flow schematic of visual tactile sensor mechanism.

The system installation employed for the vision-based tactile sensor is a combination of multiple systems, such as a motion actuator with a tactile sensor test bench, motion controller, and control personal computer (PC), as depicted in Figure 3a.

- Motion actuator with vision tactile sensor bench: The motion actuators are used in the test bench to facilitate the motion along the linear (XYZ) and rotational ($R_{x}R_{y}$) axis. The contact shaped tool is activated through actuators in order to make contact with the elastic tactile tip which has a camera fixed inside it.
- Motion controllers: The motors are controlled using the motion controllers which indeed act as a bridge between the motion actuators and control PC. This motion controller considers all the parameters, such as force, contact position, and angle, so that the motion exhibits the desired outcome as expected.
- Control PC: The control PC is a general personal computer with a LabVIEW GUI which acts as an activity log of the motions, controls, and data acquisition/processing center for the whole system installation. The training/testing data is collected from the test bench stereo camera setup via a USB port. Then, the LabVIEW software is used to accumulate the data with corresponding tactile control parameters for network training/testing.

All these subsystems gather with an intercommunication mechanism to exhibit the overall system flow schematic. The force gauge and the tactile sensor come into contact to exhibit a deformation on the elastic tactile tip, which is thereby recorded as a pattern by the stereo optical system. This mechanism is then controlled, regulated by the motion controller, and a control PC with processing software as a whole. This flow schematic of the visual-tactile sensor mechanism is shown in Figure 3b.

### 3.2. Development of Tactile Fingertips

#### 3.2.1. Process of Making Tactile Fingertips

**Figure 4:**
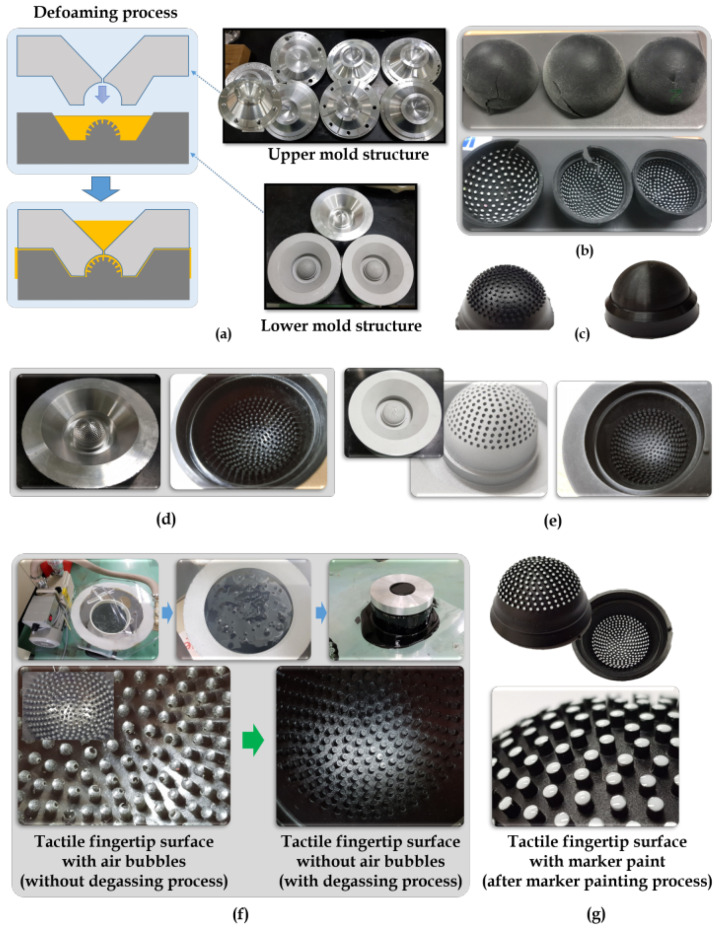
Making of tactile fingertips: (**a**) Defoaming process with upper mold and lower mold structures. (**b**) Fingertips produced from 3D printing process. (**c**) Fingertips produced from defoaming injection mold process. (**d**) Mold injection causes surface light reflection. (**e**) Sanding the mold surface reduced the light reflection. (**f**) Vacuum degassing process. (**g**) Marker painting process.

Although there are many ways to make tactile fingertips, this study proceeded with an injection mold technique with the defoaming process, as shown in Figure 4a. Before the injection mold tips, several 3D printing processes were employed to produce tactile fingertips. Yet, they all did not withstand the stress and were torn apart, as shown in Figure 4b. The defoaming process with injection mold structures (upper, lower) used helped in withstanding the elastic stress imposed by repeated force gauging. However, there are some practical issues involved in the process of making the tactile fingertips. One such issue is the problem of surface light reflection on the inside of the tactile fingertip. The injection mold process which was opted by this study posed this issue of light reflection, as shown in Figure 4d. The major concern is that these light reflections will overshadow the deformation patterns inside the tactile fingertips. This could lead to inappropriate optical imagery captured by the stereo camera inside the fingertip. Therefore, the process of sanding was sequentially carried out on the mold surface after the injection process to reduce the light reflections, as shown in Figure 4e.

The reliability of the tactile fingertips is crucial in this study as they are often exposed to a repetitively pressing process to collect the force, contact position, and other tactile-based sensor data. Accordingly, the reliability of the tactile tips can be categorized into physical and visual terms.

- Physical: The tactile tip must sustain the repetitive stress and must exhibit the same tactility throughout the sensor data acquisition. But, often, the insides portion of the tactile tip severely suffers from air bubbles. This problem was encountered in this study, and it was successfully resolved using the process of vacuum degassing of the tactile tip while manufacturing it. This process is shown in Figure 4f, and it efficiently reduced the air bubbles and offered better endurance to the tactile tips.
- Visual: The visual reliability of the tactile tip was improved by the marker painting process, as shown in Figure 4g, which helped in the recognition of deformation patterns visually. Initially, a white paint is to mark the markers on the surface of the sensor. During the durability test, the markers were not compatible with the tactile sensor rubber material. Therefore, the marker painting is done using the same rubber material but with white color for easy recognition.

#### 3.2.2. Tactile Fingertip Sensor Design Aspects

The tactile sensor fingertip specifications considered in this study are stated below in Table 2.

In the process of making the tactile fingertips, an ablation study was put forward to analyze certain practical aspects, such as which material should be used to make the tips, what should be the thickness of the tactile tip, etc. These questions were investigated using a proper ablation study in terms of tactile touch sensitivity and tactile stability. The force-displacement characteristic plot was constructed to analyze the effect of shore hardness, i.e, surface hardness of the material and the thickness of the material. The shore hardness is often measured using shore hardness scale or durometer shore hardness scale, denoted as “Shore 00” hardness scale [44]. For example, if a material is very soft, such as gel, then the shore hardness scale will be shore 05; and, if it is a hard rubber, such as shoe heel, then the shore hardness scale will be shore 100.

**Figure 5:**
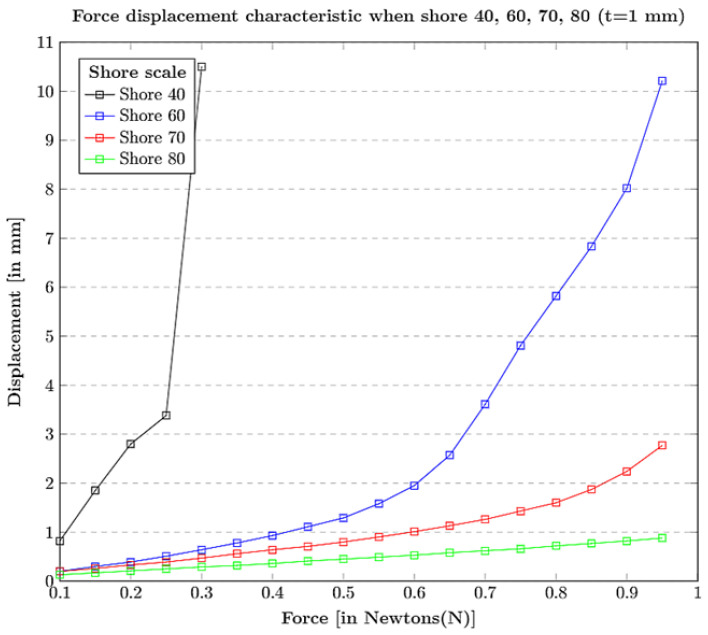
Selection of tactile fingertip material based on shore hardness (surface hardness): Force displacement characteristic when shore hardness is 40, 60, 70, 80 for t = 1 mm.

**Figure 6:**
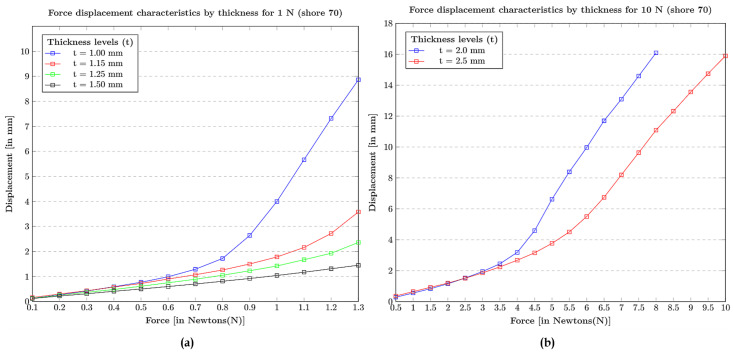
Choosing the thickness of the tactile fingertip: (**a**) Force displacement characteristics by thickness at 1 N for shore 70. (**b**) Force displacement characteristics by thickness at 10 N for shore 70.

- Shore hardness (surface hardness): The tactile materials with a standard thickness t = 1 mm are considered with different shore hardness scales = 40, 60, 70, 80. The force-displacement characteristic plots can be observed in Figure 5 where, with the increase in the force, the tactile tip with shore hardness 40 is easily displaced losing its linearity in terms of elasticity, i.e, the tip with shore hardness 40 is too weak to be used as an elastic body at force 1 N. Similarity, with the increase in the force, the tactile tip material with shore hardness 60 seem to have similar displacement characteristics, like the shore hardness 40 material, but a bit linear. In contrast, the comparison between shore hardness 70 and 80 resulted in choosing the optimal shore hardness of 70 for the study experiments because shore hardness 80 is insensitive to be an elastic material with linearity at various force steps.
- Thickness (elastic stability): The materials with an optimal shore hardness range 70, were chosen. Then, the thickness t = 1 mm, 1.15 mm, 1.25 mm, 1.50 mm were investigated with an applied force of 1 N, as shown in Figure 6a, and thickness t = 2.0 mm, 2.5 mm were investigated with an applied force of 10 N, as shown in Figure 6b. At an applied force 1 N, the material with thickness of t = 1 mm is suitable for the deformation of 4 mm, and all the rest, t = 1.15 mm, 1.25 mm, 1.50 mm, cannot be used if the expected deformation is 4 mm or higher. For an applied force of 10 N, material with thickness t = 2 mm collapsed when the force reached 7 N, but the material thickness t = 2.5 mm is stable at 10 N. This ablation study facilitates the choice of better tactile fingertips for the experiments.

### 3.3. Stereo Camera System

**Figure 7:**
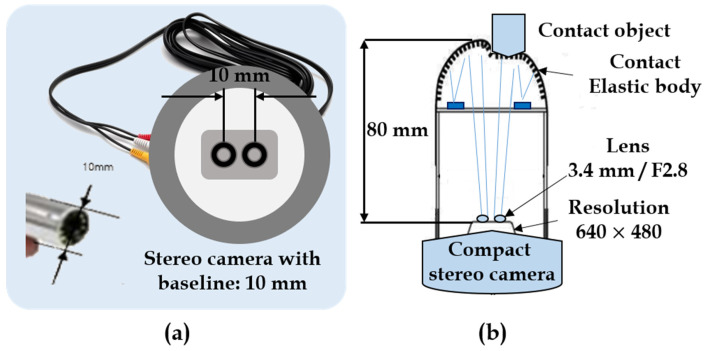
Stereo camera system: (**a**) Stereo camera with baseline of 10 mm. (**b**) Compact stereo camera attached to tactile fingertip.

The stereo camera system is fixed at the bottom of the tactile elastic tip to capture the deformations caused by the tactile contact. To acquire better image data from the tactile mechanism, the choice of the stereo system has been made. The stereo camera captures both the right image and left image of the deformation and transfers the image data to the control PC for training/testing purposes. The design setup of the stereo camera system used in this study is shown in Figure 7 below.

The visual-tactile sensor system heavily relies on this stereo camera system for the inference in real-time. Therefore, the system must be compact, memory-friendly, and power-efficient. The stereo setup used in this study is compact such that its stereo baseline between the right and left camera lens is a mere 10 mm distance with optimal industrial standard size of 640 × 480, which is efficient in terms of memory and power consumption. Nevertheless, other image resolutions, such as 1280 × 720 with 30 fps, 640 × 360 with 30 fps, and 320 × 240 with 30 fps, were also examined. The design aspects of the stereo camera system employed in the experiments are stated in Table 3.

### 3.4. Deep Learning Methodology

**Figure 8:**
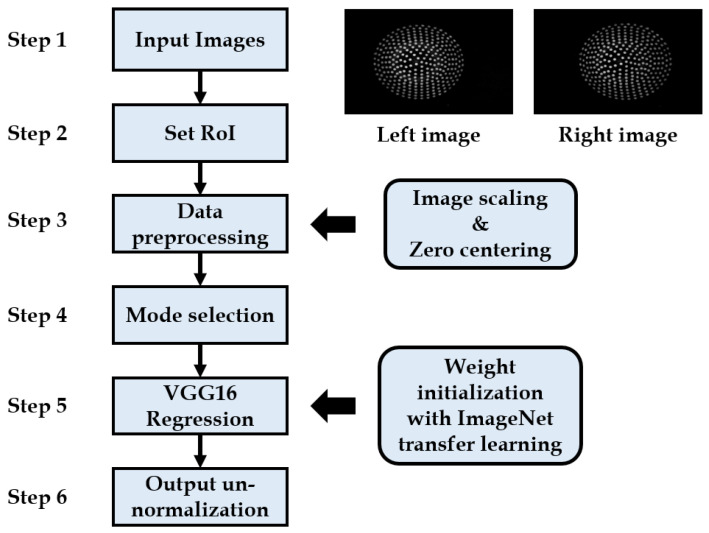
Flowchart schematic of transfer learning applied on the images acquired from tactile stereo camera setup.

The deep learning-based contact position and force measurement algorithm is divided into six steps, which are shown in Figure 8 and described in detail below. The stereo image pair consisting of the deformation pattern of the elastic tactile tip serves as an input for the algorithm. Both the right and left images have the same deformation pattern but from a different perspective with a baseline of 10 mm in between both imagery. Data handling, pre-processing, and transfer learning are the crucial steps involved in the learning algorithm.

#### 3.4.1. Region-of-Interest (ROI) and Mode Selection

**Figure 9:**
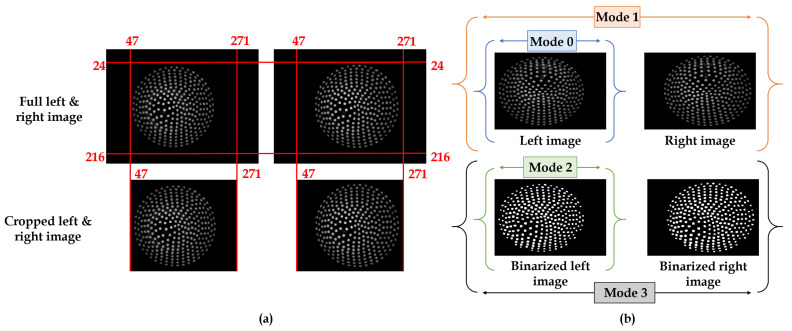
Pre-processing: (**a**) Cropping the input data through Region-of-Interest (ROI) setting for the stereo image pair. (**b**) Types of modes.

The ROI setting was carried out to enable memory management and save the processing power of the GPU. While considering a single input image of 3 channels (RGB) with dimensions 640 × 480, the video input from left and right cameras via acquisition equipment in terms of height, width, channels is 480, 640, 3. Therefore, it is essential to design a region of interest that suits both left and right images. Accordingly, a manual ROI area is calculated as per the video input specifications to be the same for the whole stereo pair data. The ROI setting for the Row is: from 24–216 pixels; ROI setting for the Column is: 47–271 pixels; and the cropped area size is (192, 224), which can save GPU memory to the maximum. The ROI design is shown in Figure 9a, which is the same for both the right and left images.

The mode selection is a customized procedure designed to test the best possible input feed to insert into a neural network for better results. This procedure involves the selection of the data as per different modes, as shown in Figure 9b, and then feed them into the neural network as input. Although 4 modes were put-forward, the mode that performs well during training (mode 1) will only be considered for the inference.

- Mode-0: This mode will only consider the left image from the stereo pair as an input to the neural network.
- Mode-1: This mode will concatenate left and right gray images per channel and input them to the neural network.
- Mode-2: This mode will consider the left image binarized to enhance lighting and feed it to the neural network as input.
- Mode-3: This mode will concatenate the left and right images binarized for each channel to enhance lighting and feed it to the neural network as input.

#### 3.4.2. Zero Centering and Scaling

The combination of image data with a coupled tactile sensing data must be well fused and analyzed for the network to train on the insights of the data, although the video input stereo images received from the equipment are pre-processed by cropping and setting a specific ROI to optimize the memory and power. However, there is also a need to further process the image data such that the fusion of tactile data which is in terms of force, contact location, contact angle, etc., can be possible. In other words, for the deep learning network to converge well during the learning process, unit8 (an unsigned integer) [0–255] image is scaled to [0, 1] and normalized to [−1, 1] by zero-centering. The reason for performing zero centering and scaling is because the attribute to be predicted is different in terms of units and ranges, such as displacements along X, Y, Z, which are (in mm) Force (in N), $R_{a}$ (in degree). Therefore, the zero centering and scaling is essential for the network to learn the insights of the image data in correspondence with the tactile parametric data. The zero centered and scaled data (${\tilde{x}}_{ij}$) will be a function of original data ($x_{ij}$) normalized between the minimum ($min_{j}$) and maximum points ($max_{j}$), as shown in:

(1) $${\tilde{x}}_{ij}=\frac{x_{ij}-min_{j}}{max_{j}-min_{j}},$$

where *i* is the data index, *j* is the attribute index, $x_{ij}$ is the *j*th attribute of the *i*th data, $max_{j}$ is the maximum value of the *j*th attribute of the training data, and $min_{j}$ is the minimum value of the *j*th attribute of the training data.

#### 3.4.3. Network Architecture

**Figure 10:**
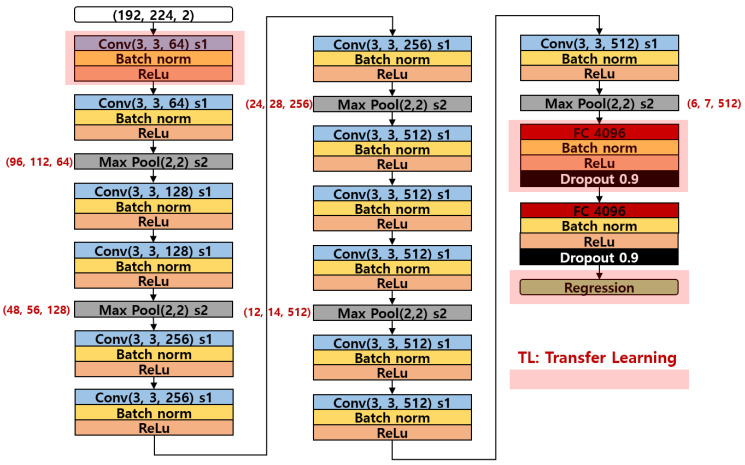
Network architecture of VGG16 regression model employed in the study.

The convolutional neural network model used in this study was adopted from the well-known VGG16 structure. Often, the VGG16 model structure is exploited to acquire better accuracy for the object classification tasks in computer vision and AI domains. However, the task that this study has to accomplish is to predict the continuously varying parameters, such as force, contact position, angle, etc. These parameters are indeed the continuous values that cannot be modeled into a classification task. The customized convolutional neural network model consists of total 16 deep layers, including the input layer. The input layer is fed to the neural model, and the input must pass through 16 deep layers, along with 5 max pooling layers. The first 2 layers of the network consist of 64 channel convolution filters of size 3 × 3 with stride 1 followed by a batch normalization and a Rectified Linear Unit (ReLU) activation function. The max pooling of size 2 × 2 with a stride 2 is used after the second convolution layer. The max pooling used throughout the model has a standard configuration of size 2 × 2 with a stride 2. The next 2 convolution layers use a 128 channel convolution filters of size 3 × 3 with stride 1 followed by a batch normalization, ReLU activation function. The max pooling layer is used after the fourth convolution layer. The next 3 convolution layers consists of 256 channel convolution filters of size 3 × 3 with stride 1 followed by a batch normalization, ReLU activation function. The max pooling layer is used after the seventh convolution layer. The next 6 convolution layers contain 512 channel convolution filters of size 3 × 3 with stride 1 followed by a batch normalization, ReLU activation function. The max pooling layer is used after tenth and thirteenth convolution layers. The last two layers are dense fully connected layers with 4096 units each. To prevent the overfitting, a dropout of 0.9 was used. The total output from the fully connected dense layers is used for the regression purpose, as shown in Figure 10.

### 3.5. Contact Area Estimation

**Figure 11:**
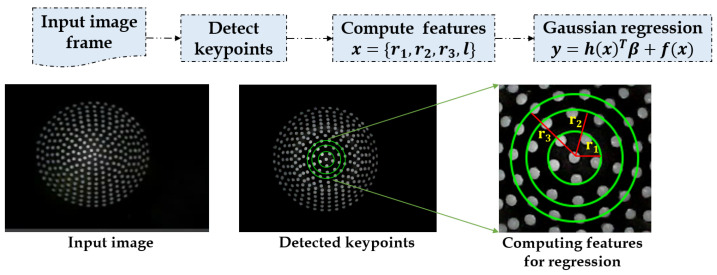
Flow schematic of 2D contact area estimation process.

The contact area estimation is designed to use the images acquired from the stereo camera to estimate the 2D contact area using naive computer vision methodology, as depicted in Figure 11. The contact area estimation was put forth to analyze the effect of sensor shapes on the contact area. Similarly, the ground truth of the known sensor tips were employed to investigate the errors in the estimated area.

The input frame is used to identify the deformations on the elastic tip, and the keypoints are detected using image processing techniques, such as image segmentation and blob analysis [45]. These keypoints are then used to calculate the radii (*r*) depending upon the shape of the contact tool (*l*) used. The features are then used as a dataset to apply Gaussian regression to get the contact area, as shown in:

(2) $$x=\left\{r_{1},r_{2},r_{3},l\right\},$$

where $r_{1},r_{2},r_{3}$ are the radii from center to the keypoints; *l* is the shape of the contact tool, such as circle, square, and hexagon; and *x* is the feature vector.

(3) $$y=h{\left(x\right)}^{T}\beta +f\left(x\right),$$

where f(x) is the function from zero mean Gaussian Process, h(x) is the transform function, square and hexagon, β is the hyper parameter, and f,h are learned in the training process.

## 4. Experiments and Evaluations

### 4.1. Dataset Used

**Figure 12:**
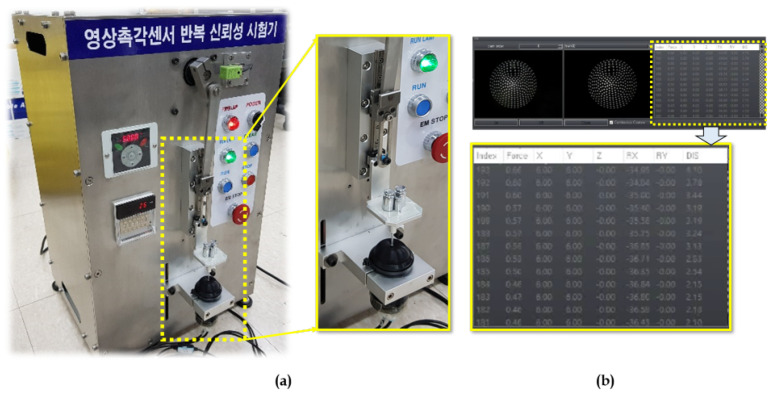
Data acquisition procedure for training and testing scenarios: (**a**) Instrument to conduct experiments. (**b**) LabVIEW GUI for collecting data under various motions ($X,Y,Z,R_{x},R_{y}$).

The tactile contact force gauge equipment used for the collection of data is shown in Figure 12. The data retrieved from the equipment is used to construct the training, validation, and testing dataset. The collected data is transferred to the control PC via a USB port, which is then processed using LabVIEW GUI on the PC. Figure 12b shows the log of all the sensor data ($X,Y,Z,R_{x},R_{y}$) recorded simultaneously with the stereo images. This GUI will have the timestamp of the data which is used to fuse the tactile data with the stereo images. Various shaped contact tools were employed in the experiments to get the force and contact location.

The dataset used in the network training is divided into training, validation, and testing which is shown in Table 4. Data01 and Data02 are two splits of the data which are separated as per the sensor size (thin, thick). Each split of the data is internally divided into training, validation, and testing. In Table 4, the training, validation, and testing are depicted (per point) because this data is acquired by applying diverse force levels starting from 0.1 N to 1 N with an interval of 0.1 N. Therefore, for each force applied point, the acquired image stereo pair count is given Data01 containing (2∗3380) training samples, (2∗1680) validation samples, and (2∗1690) testing samples. Similarly, Data02 containing (2∗2730) training samples, (2∗910) validation samples, and (2∗910) testing samples. On a whole, the total images used in the training are 122,200, validation are 51,800, and testing are 52,000 samples.

### 4.2. Training Details

The training is carried out with several aspects inculcated into the data, such as considering different data splits with various modes under diverse sensor sizes, such as thin and thick.

**Figure 13:**
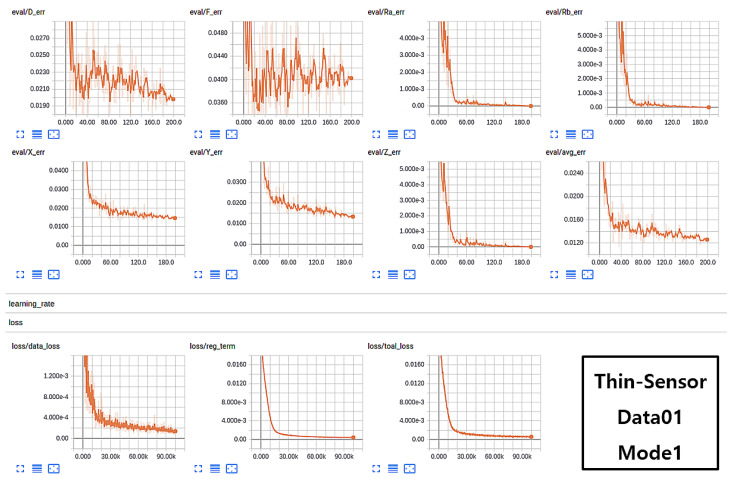
Successful case scenario of network training on thin sensor data (Data01) in the form of Mode1.

**Figure 14:**
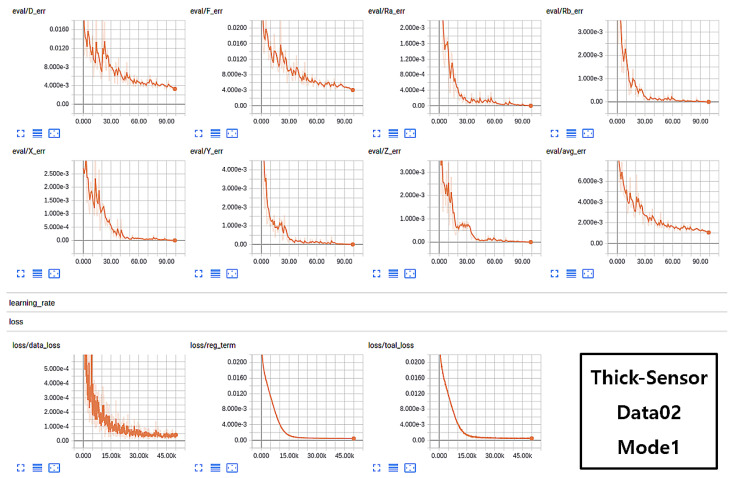
Successful case scenario of network training on thick sensor data (Data02) in the form of Mode1.

The training sessions were carried out on Data01 and Data02 and evaluated using the validation data for each iteration. The approach of validation is carried out to prevent the network from overfitting and the best model is then saved as a final trained network. The models were also trained under various sensor sizes, such as thin sensor and thick sensor, with induced forces of 1 N and 10 N, respectively. The training scenario and trained model on Data01 with mode1 considering stereo pair (both right and left images) for training acquired with a thin sensor exhibited better accuracy. The graphs in Figure 13 represent various training aspects, such as validations over Force (*F*), Displacement (*D*), Position (X,Y,Z), and Rotations ($R_{x}\;and\;R_{y}$). The seven charts in the figure above are the results of experiments on validation data for 7 attributes [$F,D,X,Y,Z,R_{x},R_{y}$]. Avg err is the average error of all 7 attributes, which should be as low as possible for a better-trained method. The three graphs in the bottom row of Figure 13 represent data loss, regularization term, and total loss in the learning process. Similarly, the analysis of the training process using Data02 split with Mode1 samples is shown in Figure 14.

### 4.3. Testing Evaluations

**Figure 15:**
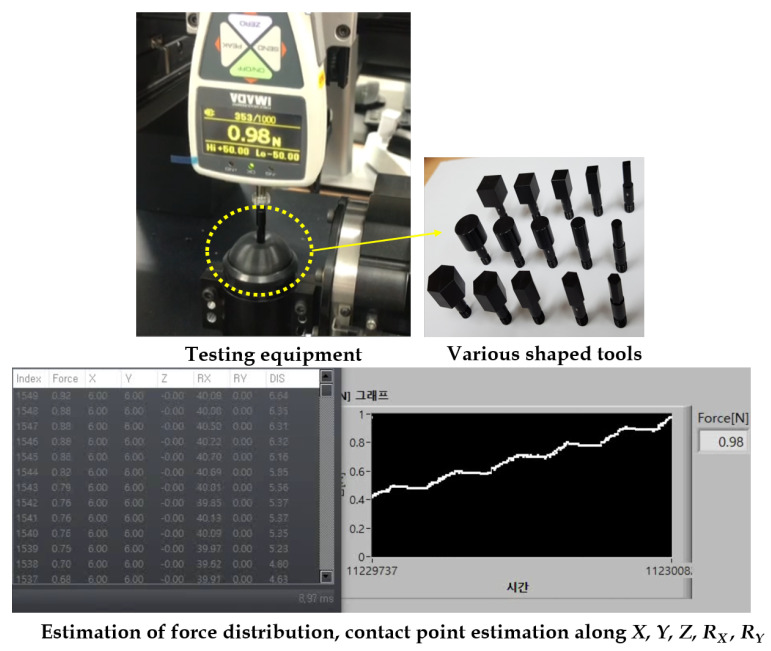
Testing scenarios and outcomes.

The performance evaluations were carried out for all the testing scenarios (contact force, contact position displacement, contact position rotation, contact area estimation) using several metrics, such as error rates, full scale output, average error, etc. The testings were carried out exhaustively using various shaped tools, force levels, sensor sizes, and displacements, as shown in Figure 15.

#### 4.3.1. Testing Scenario-1: Force Distribution Estimation

The testing scenario of the force distribution estimation is carried out 10 times with each time 10 steps ranging from 0.1 N to 1 N. The testing performance of the trained system in predicting the force (in N) correctly is evaluated by error calculation between the applied force and estimated force value. The evaluation metric named Full Scale Output (FSO) in % is calculated to quantify the performance of the predicted force, as shown in:

(4) $$FSO\left[\%\right]=\frac{{\left|\left(F_{in}-F_{pred}\right)\right|}_{\max}}{{\left|F_{in}\right|}_{\max}}*100,$$

where $F_{in}$ is the applied input force in Newtons (N), $F_{pred}$ is the predicted force by the trained neural network in terms of Newtons (N), ${\left|(F_{in}-F_{pred})\right|}_{max}$ is the maximum value of the difference between actual and predicted force, and ${\left|(F_{in})\right|}_{max}$ is the maximum value of the applied force.

#### 4.3.2. Testing Scenario-2: Contact Point (Displacement) Estimation along Linear *X*-axis, *Y*-axis, an *Z*-axis

The contact point position (displacement) along the *X*-axis, *Y*-axis, and *Z*-axis is estimated by the trained neural network, and the testing accuracy is calculated by the error between the original displacement along X,Y,Z and estimated displacement along X,Y,Z. The testing evaluations were carried out as follows:

- Along *Z*-axis: The force is applied in *Z*-direction from 0.1 N to 1 N with 0.1 N interval such that total 10 tests were conducted. The difference between the original position along *Z*-axis and the estimated one is recorded as the error and an average error over 10 tests is calculated to evaluate the performance of the prediction.
- Along *X*-axis: For evaluating the displacement along *X*-axis, the force is applied in intervals of 0.1 N from 0.1 N to 1 N with 1-mm displacement step along the *X*-axis keeping the *Y*-axis displacement as 0. Therefore, the testing is done for (X= −6 mm ∼+6 mm, with 1 mm step interval, total 13 points, constant Y=0). The difference between the original position along *X*-axis and the estimated one is recorded as the error and an average error over 13 points is calculated to evaluate the performance of the prediction.
- Along *Y*-axis: For evaluating the displacement along *Y*-axis, the force is applied in intervals of 0.1 N from 0.1 N to 1 N with 1-mm displacement step along the *Y*-axis keeping the *X*-axis displacement as 0. Therefore, the testing is done for (Y= −6 mm ∼+6 mm, with 1 mm step interval, total 13 points, constant X=0). The difference between the original position along *Y*-axis and the estimated one is recorded as the error and an average error over 13 points is calculated to evaluate the performance of the prediction.

The evaluation metric used to evaluate these displacements along XYZ axis is calculated to quantify the performance of the predicted force using mean absolute error (MAE), as shown in:

(5) $$MAE=\frac{1}{n}\sum_{1}^{n}\left|d_{\mathrm{orig}}-d_{\mathrm{est}}\right|,$$

where N is the number of tests/points performed, $d_{orig}$ is the original displacement values, and $d_{est}$ is the estimated displacement values by the neural network.

#### 4.3.3. Testing Scenario-3: Contact Angle Estimation along Rotational $R_{xy}$ axis

The contact angle estimation along the rotational axis $R_{x}R_{y}$ is evaluated using the 10 tests when force is applied from 0.1 N to 1 N with 0.1 N interval. The tests were performed such that the original angle along the rotational axis $R_{x}R_{y}$ is set to 45${}^{\circ }$. The mean absolute error (MAE) is calculated between the estimated and original angle, as shown in:

(6) $$MAE=\frac{1}{n}\sum_{1}^{n}\left|R_{a_{\mathrm{orig}}}-R_{a_{\mathrm{est}}}\right|,$$

where N is the number of tests/points performed, $R_{a_{orig}}$ is the original angle 45${}^{\circ }$, and $R_{a_{est}}$ is the estimated angle values by the neural network. The sensor is rotated along the × axis and *Y*-axis to a calibrated ground-truth of 45${}^{\circ }$, which is considered to be the ground-truth for the rotational test scenarios. The system installation heavily influences the performance due to the rotational motions. Therefore, a constructive ground-truth of 45${}^{\circ }$ is calibrated so as to prevent the system installation issues.

#### 4.3.4. Testing Scenario-4: 2D Contact Area Estimation

The testing for the 2D contact area estimation was carried out using various shaped contact tools that are used to contact the elastic tactile tip. The contact area estimates are derived from the Gaussian regression process described earlier. The ground truth (GT) of the contact area is fixed when the tool is used to make contact, and it is used to calculate the error between the estimated and GT. The evaluation of the performance is calculated by error rates in (%), as shown below:

(7) $$\%Error.rates=\frac{\left|CA_{GT}-CA_{est}\right|}{CA_{GT}}*100,$$

where $CA_{GT}$ is the ground truth contact area, and $CA_{est}$ is the estimated contact area from the Gaussian regression.

## 5. Results and Discussions

### 5.1. Force Distribution Estimation

The force estimation carried out using the trained network was validated by using 10 different tests among which each test was recorded within a force range of 0.1 N ∼1 N. The estimation errors were recorded in N and were used to calculate the FSO (%) scores. The force estimation errors which were recorded on all 10 tests are depicted in Table 5. The overall average on a whole (10 tests) is around 0.022 N which is accurate for the system to rely on the estimations for future predictions.

**Figure 16:**
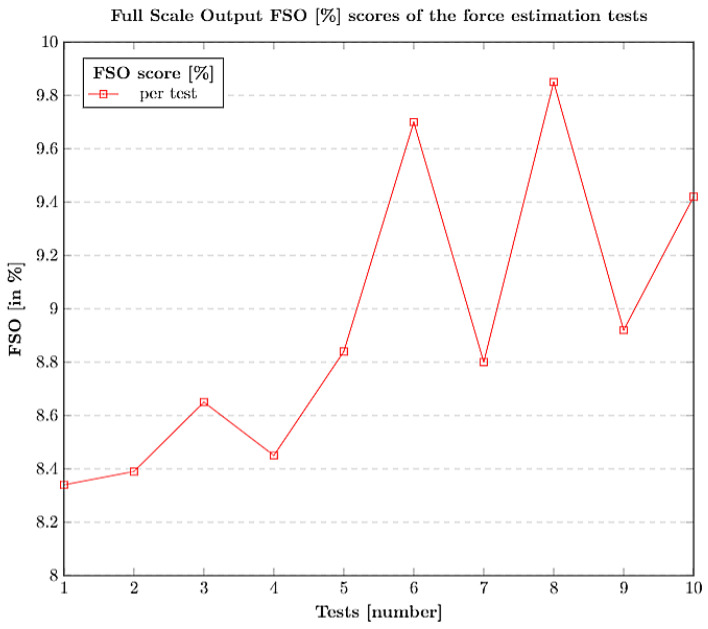
Full Scale Output (FSO) (%) output scores for force estimation tests.

The FSO (%) scores of all the 10 tests are plotted in Figure 16, and the average FSO (%) score seemed promising, within the force range of (0.1 N∼1 N). The entire data samples, and their corresponding estimation errors for each iteration (test) and their averages, FSO (%) scores, etc., are presented in Table A1 in Appendix A.

### 5.2. Contact Position Estimation w.r.t X,Y,Z Axes

**Figure 17:**
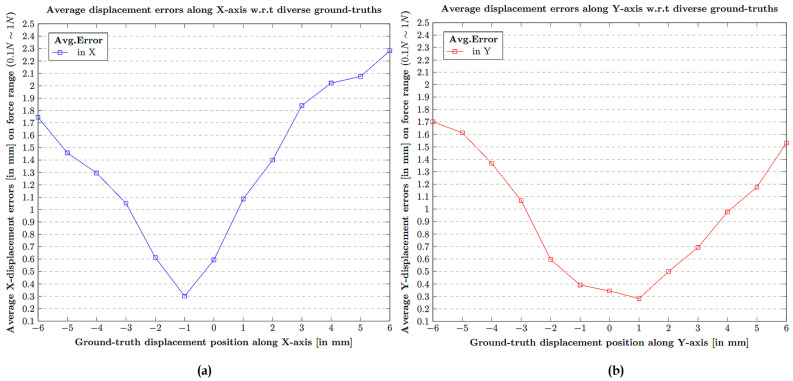
Average displacement error in X,Y contact position w.r.t corresponding ground-truth over 13 points (−6 mm∼+6 mm): (**a**) Average X-displacement errors (in mm). (**b**) Average Y-displacement errors (in mm).

The contact position displacement errors were calculated and are estimated for each force measure ranging from (0.1 N∼1 N) with respect to each ground truth value in × and also in Y spanning from (−6 mm∼+6 mm). The displacement errors covering all the possible test ranges are clearly depicted in Table A2 and Table A3 in Appendix A. Table A2 in Appendix A represents the test results in terms of displacement error in the contact position along the X-axis. Similarly, Table A3 in Appendix A represents the test results in terms of displacement error in the contact position along the Y-axis. The average error displacement readings in correspondence to the 13 point ground truth values (−6 mm∼+6 mm) over the force (0.1 N∼1 N) is shown in Figure 17.

**Figure 18:**
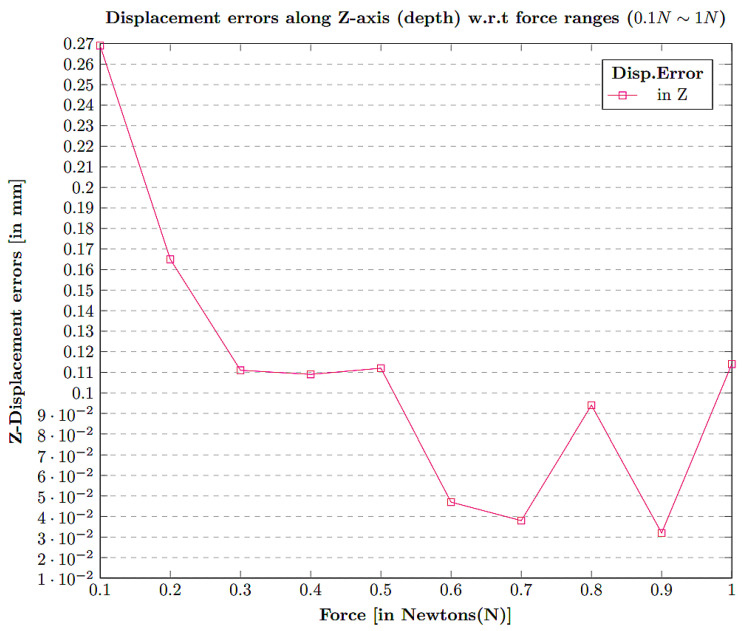
Displacement error in *Z* contact position w.r.t diverse force ranges 0.1 N∼1 N.

The contact position displacement error along the Z-axis was calculated by evaluating the estimation values of Z-axis displacement for a given force value ranging from 0.1 N∼1 N. The overall estimation error w.r.t force values are depicted in Figure 18.

The results of the contact position displacement estimation in the X, Y, Z axes revealed the performance of the network in predicting the position estimates. The estimation error along the × and Y axes is greater than that in the Z-axis. The reason for that the motion in × and Y indeed requires Z, as well. Therefore, even while acquiring × and Y data, the underlying Z data keeps on feeding into the system.

### 5.3. Contact Angle Estimation w.r.t Rotational $R_{xy}$ Axis

**Figure 19:**
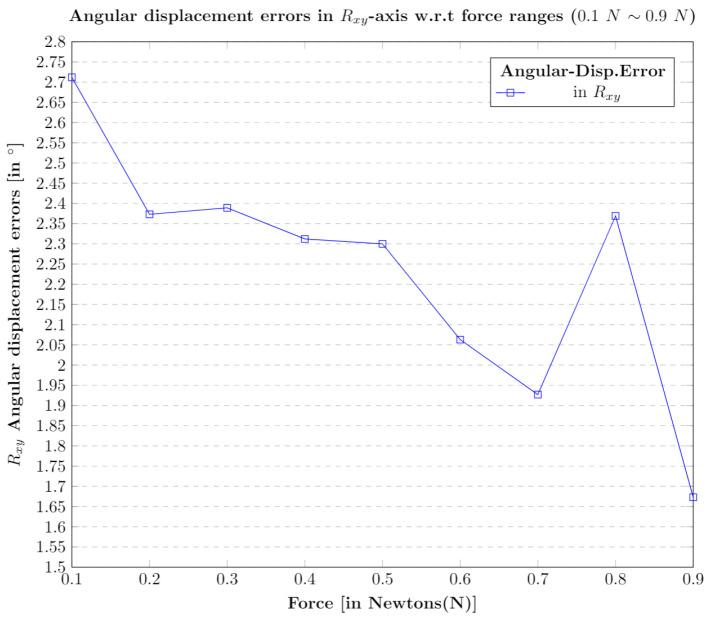
Angular displacement error in $R_{xy}$ axis w.r.t diverse forces (0.1 N∼0.9 N).

**Figure A1:**
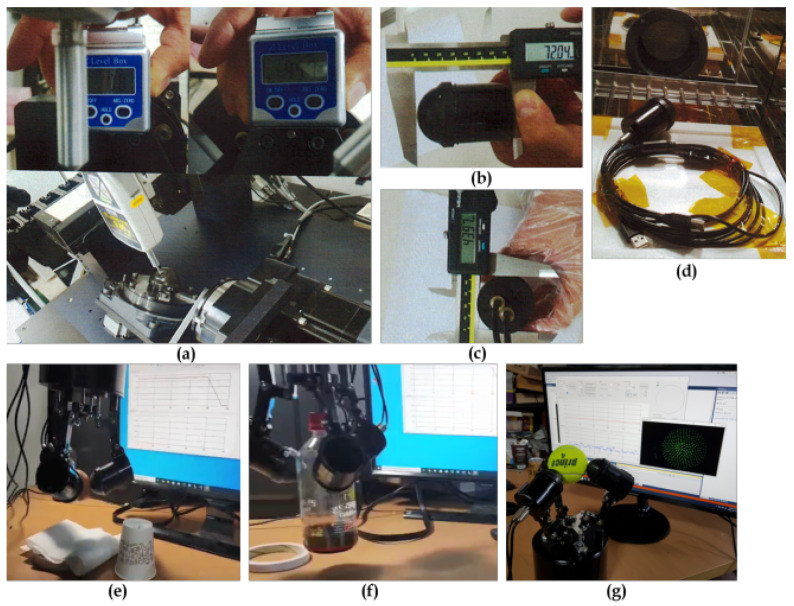
Tactile sensor-related aspects: (**a**) Stable calibration of tactile sensor at an angle of $45^{\circ }$. (**b**) Measuring the height of tactile sensor (72 mm) using vernier caliper. (**c**) Width of tactile sensor (44 mm). (**d**) Tactile sensor camera. (**e**) Vision-based tactile sensor used in a robotic arm grasping a raw egg. (**f**) Lifting and placing a bottle. (**g**) Grasping a tennis ball.

The contact position estimation in terms of angular displacement was calculated through a series of tests within the force range of 0.1 N∼1 N. The sensor is rotated with a fixed angle of $45^{\circ }$, and the tests were performed through contact tool in touch with the sensor which is inclined. The reason for the calibrated ground truth fixed angle of $45^{\circ }$ is discussed in Appendix A and is depicted clearly in Appendix A, Figure A1a.The trained neural network was able to predict/estimate the angular displacement in the contact position. The results of the estimated displacement w.r.t each force value is depicted in Figure 19.

### 5.4. Contact Area Estimation

**Figure 20:**
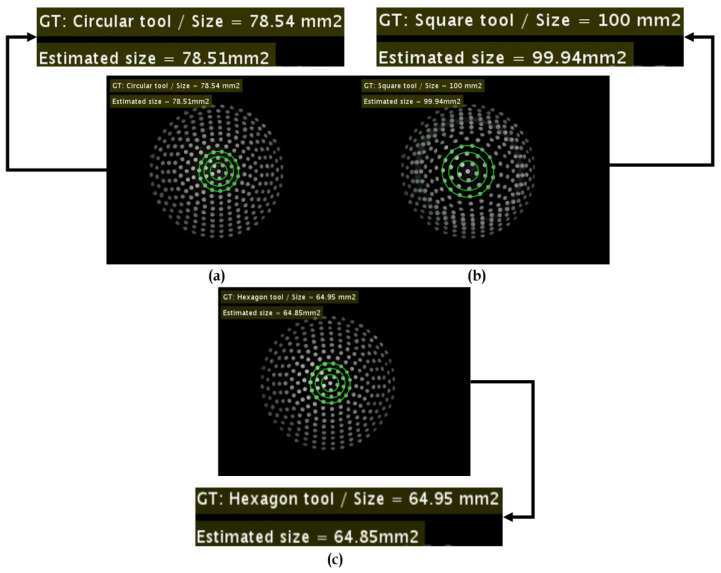
Estimation of 2D contact area: (**a**) Contact area estimation w.r.t circular tool. (**b**) Contact area estimation w.r.t square tool. (**c**) Contact area estimation w.r.t hexagonal tool.

The contact area estimation is carried out using the image processing algorithms and Gaussian regression. The estimated contact area is cross checked with the ground truth in correspondence with various contact shaped tools. The corresponding results are reported in Table 6. Figure 20 illustrates the estimation errors w.r.t circular tool with ground truth (GT=78.54 mm${}^{2}$), square tool with (GT=100.00 mm${}^{2}$) and (GT=64.95 mm${}^{2}$).

There were different samples considered for each tool shape for the testing, such as circular (n=20), square (n=18), and hexagonal (n=18). The results suggest that the estimation of the contact area in the case of hexagonal tool seemed more prone to errors. However, on a whole, the total average error is 1.429% on all the contact shaped tools.

## 6. Conclusions

This work reports the usage of deep learning-based visual-tactile sensor technology for the estimation of force distribution, contact position displacement along *X*, *Y*, *Z* directions, angular displacement along $R_{x}R_{y}$ direction and contact area. The current study also reports the design aspects, such as choice of the thickness and materials used for the tactile fingertips, encountered during the development of the tactile sensor. The image acquisition was carried out using a compact stereo camera setup mounted inside the elastic body to observe and measure the amount of deformation by the motion and input force. The transfer learning has been employed using the VGG16 model as a backbone network. Several tests were conducted to validate the performance of the network in estimating the force, contact position, angle, area using calibrated ground-truth values of force range 0.1 N∼10 N, position range −6 mm∼+6 mm, fixed angular value of $45^{\circ }$. The tests were also carried out using thick, thin tactile sensors with various shapes, such as circle, square, and hexagon, along with their ground truth areas. The results determine the average estimation errors for force, contact position in X, Y, Z, contact angle and contact area are 0.022 N, 1.396 mm, 0.973 mm, 0.109 mm, $2.235^{\circ }$, and 1.429%, respectively. However, the future work should include improvements handling system stability in terms of tactile sensor sensitivity w.r.t reference axes and movements in the vicinity. Nevertheless, the results reported in the study corresponds to the significance of the visual-based tactile sensor using deep learning as an inference tool.

## Figures and Tables

**Table 1:** Insights of traditional and learning-based visual tactile sensing methods.

Research Study	Methodology	Tactile Properties	Key Aspects/Limitations
Lepora et al. [27]	Bayesian perception	Localization (internal displacement)	40-fold accuracy compared to traditional tactile sensor
Ito et al. [28]	Adaptive selection and compensation of dot positions	Slippage degree multidimensional force object contact	Depends on position, measurements of dots (tuning is easy)
Yang et al. [29]	Magic finger optical touch sensor	Contact location force and texture	Can sense the touch finger XY-footprint (like optical mouse)
Corradi et al. [30]	Object recognition (vision + touch)	Object shape/texture	Vector concatenation object label posterior
Piacenza et al. [32]	Elastomer Light transport mechanism	Contact Localization and Indentation depth prediction	Exhibits submillimeter accuracy 20 mm by 20 mm active sensing area
Johnson et al. [33]	Surface reconstruction (photometric stereo)	Texture and shape	In addition, called as 2.5D texture scanner
Johnson et al. [34]	Microgeometry using Elastomeric Sensor	Surface geometry	Can only handle shallow relief geometry
Yuan et al. [35]	Object hardness with GelSight touch sensor	Fine texture, contact force and slip conditions	Infer object hardness without prior knowledge
Kroemer et al. [36]	Dynamic tactile sensing using weak pairing (vision + tactile samples)	Visual shape and surface texture	Machine Learning with lower dimensional representation of tactile data
Meier et al. [37]	Tactile DeepCNN for Online Slip and Rotation Detection	Classify contact state Distinguish rotational and translation slippage	Final classification rate is >97% Feasible for adaptive grasp control
Chuah et al. [38]	Least Squares ANN improving shear force with better optimization	Normal and Shear tactile force	Better convergence with Multi-input, multi-output function approximator
Kaboli et al. [39]	Probabilistic active tactile transfer learning	Surface texture, stiffness, and thermal conductivity	72% discrimination accuracy only one training sample (on-shot-tactile-learning)
Gandarias et al. [40]	Custom CNN (TactNet) for object recognition with RGB pressure images	Contact objects Identify tactile pressure	Used 8 transfer learning networks, 3 TactNet scratch training
Sato et al. [42]	Compact finger-shaped GelForce sensor for surface traction fields	Measuring distribution of force vectors or surface traction fields	Small size with linearity of force <4 N with refresh rate 67 Hz
Sferrazza et al. [43]	Commercial force tactile sensor images are matched to groundtruth data for DNN training	Measuring contact force and contact center of the sensor’s surface	Refresh rate of 40 Hz, performance is dependent on reference axes alignments
Current study	Transfer learning-based CNN training using tactile sensor images matched with groundtruth	Measuring contact force contact position in $X,Y,Z,R_{x},R_{y}$ and contact size in mm${}^{2}$	Refresh rate of 30 Hz with spatial resolution of 2.5 mm and size of sensor is high than that of References [42,43] because of stereo-camera

**Table 2:** Design aspects and specifications of the tactile fingertip sensor.

Design Aspects	Specifications
Sensor surface material (including markers)	Rubber
Sensor size (width × height)	44 mm × 72 mm
Spatial resolution	2.5 mm
Refresh rate (sampling frequency)	30 Hz
No. of Protrusions	292

**Table 3:** Design aspects and specifications of the stereo camera system.

Design Aspects	Specifications
Camera resolution	640 × 480 with 30 fps
Pixel size	3 um × 3 um
Image sensor size	1/4”
Image active area	3888 um × 2430 um
Signal-to-Noise ratio	39 dB
Scan mode	Progressive
Lens module	3.4 mm/F2.8
Power	DC 5 V/150 mA
Interface	USB 2.0

**Table 4:** Dataset employed for training, validation, and testing.

Category	Training (Point)	Validation (Point)	Testing (Point)
Data01 (Left + Right)	3380 + 3380	1680 + 1680	1690 + 1690
Data02 (Left + Right)	2730 + 2730	910 + 910	910 + 910
Total (Data01 + Data02)	12,220	5180	5200
Number of images for total 10 points (0.1 N interval from 0.1 N to 1 N)	122,200	51,800	52,000

**Table 5:** Force estimation errors; Each error reading is an average of force estimation error recorded under the force range (0.1 N∼1 N) with 0.1 N interval for each test.

Test No. (1∼5)	Force Estimation Error (N)	Test No. (6∼10)	Force Estimation Error (N)
1	0.027	6	0.022
2	0.026	7	0.021
3	0.023	8	0.018
4	0.023	9	0.022
5	0.022	10	0.023
**Average Error on all 10 tests**		**0.022**	

**Table 6:** Contact area estimation w.r.t different shaped contact tools (circular, hexagonal, and square).

Shape (Contact Tool)	Number of Images (Testing)	Error Rates in (%)
Circle	20	0.597
Square	18	0.926
Hexagon	18	2.857
**Total Average Error**	56	1.429

## Data Availability

Not applicable.

## Appendix A. Supplementary Test Results

The trained neural network was employed on the series of ten tests, and the force distribution estimation errors were recorded, along with the FSO (%) score. The test results are recorded and presented in Table A1.

**Table A1:** Force distribution estimation w.r.t 10 different tests under force ranges (0.1 N∼1 N).

Test-1			Test-2			Test-3		
**Original** **Force (N)**	**Estimated** **Force (N)**	**Error** **(N)**	**Original** **Force (N)**	**Estimated** **Force (N)**	**Error** **(N)**	**Original** **Force (N)**	**Estimated** **Force (N)**	**Error** **(N)**
0.12	0.2009	0.0809	0.12	0.2014	0.0814	0.12	0.2039	0.0839
0.22	0.2776	0.0576	0.21	0.2610	0.0510	0.22	0.2857	0.0657
0.32	0.3423	0.0223	0.32	0.3336	0.0036	0.33	0.3447	0.0147
0.42	0.3983	0.0217	0.42	0.3931	0.0169	0.42	0.4113	0.0087
0.51	0.4772	0.0328	0.51	0.4828	0.0272	0.50	0.4751	0.0249
0.60	0.5726	0.0274	0.60	0.5838	0.0162	0.60	0.5844	0.0156
0.70	0.6846	0.0154	0.70	0.6929	0.0071	0.70	0.6926	0.0074
0.78	0.7696	0.0104	0.78	0.7685	0.0115	0.78	0.7736	0.0064
0.87	0.8648	0.0052	0.87	0.8715	0.0085	0.88	0.8745	0.0055
0.97	0.9748	0.0048	0.97	0.9819	0.0119	0.97	0.9769	0.0069
**FSO (%)**		**8.34**	**FSO (%)**		**8.39**	**FSO (%)**		**8.65**
**Test-4**			**Test-5**			**Test-6**		
**Original** **Force (N)**	**Estimated** **Force (N)**	**Error** **(N)**	**Original** **Force (N)**	**Estimated** **Force (N)**	**Error** **(N)**	**Original** **Force (N)**	**Estimated** **Force (N)**	**Error** **(N)**
0.13	0.2120	0.0820	0.12	0.2057	0.0857	0.13	0.2241	0.0941
0.22	0.2742	0.0542	0.22	0.2812	0.0612	0.21	0.2655	0.0555
0.33	0.3468	0.0168	0.32	0.3473	0.0273	0.33	0.3508	0.0208
0.42	0.3987	0.0213	0.41	0.3995	0.0105	0.41	0.4014	0.0086
0.50	0.4721	0.0279	0.50	0.4776	0.0224	0.50	0.4889	0.0111
0.59	0.5803	0.0097	0.60	0.5962	0.0038	0.60	0.5917	0.0083
0.69	0.6903	0.0003	0.70	0.6963	0.0037	0.70	0.7020	0.0020
0.78	0.7720	0.0080	0.78	0.7780	0.0020	0.78	0.7724	0.0076
0.87	0.8693	0.0007	0.88	0.8770	0.0030	0.88	0.8778	0.0022
0.97	0.9803	0.0103	0.97	0.9745	0.0045	0.97	0.9799	0.0099
**FSO (%)**		**8.45**	**FSO (%)**		**8.84**	**FSO (%)**		**9.70**
**Test-7**			**Test-8**			**Test-9**		
**Original** **Force (N)**	**Estimated** **Force (N)**	**Error** **(N)**	**Original** **Force (N)**	**Estimated** **Force (N)**	**Error** **(N)**	**Original** **Force (N)**	**Estimated** **Force (N)**	**Error** **(N)**
0.12	0.2054	0.0854	0.11	0.2065	0.0965	0.12	0.2074	0.0874
0.22	0.2707	0.0507	0.23	0.2686	0.0386	0.23	0.2871	0.0571
0.31	0.3271	0.0171	0.33	0.3456	0.0156	0.32	0.3438	0.0238
0.42	0.4177	0.0023	0.41	0.4052	0.0048	0.40	0.3959	0.0041
0.49	0.4761	0.0139	0.50	0.4820	0.0180	0.51	0.4784	0.0316
0.60	0.5941	0.0059	0.60	0.5985	0.0015	0.60	0.5926	0.0074
0.71	0.7154	0.0054	0.69	0.6919	0.0019	0.69	0.6958	0.0058
0.78	0.7774	0.0026	0.78	0.7737	0.0063	0.78	0.7799	0.0001
0.88	0.8811	0.0011	0.87	0.8693	0.0007	0.88	0.8801	0.0001
0.97	0.9734	0.034	0.98	0.9825	0.0025	0.98	0.9831	0.0031
**FSO (%)**		**8.80**	**FSO (%)**		**9.85**	**FSO (%)**		**8.92**
**Test-10**								
**Original** **Force (N)**	**Estimated** **Force (N)**	**Error** **Force (N)**	**Original** **Force (N)**	**Estimated** **Force (N)**	**Error** **(N)**	**Original** **Force (N)**	**Estimated** **Force (N)**	**Error** **(N)**
0.12	0.2114	0.0914	0.50	0.4868	0.0132	0.78	0.7789	0.0011
0.21	0.2643	0.0543	0.59	0.5997	0.0097	0.88	0.8777	0.0023
0.32	0.3553	0.0353	0.70	0.7088	0.0088	0.97	0.9804	0.0104
0.40	0.4039	0.0039	−	−	−	−	−	−
**FSO (%)**				**9.42**				
**Average FSO (%)**				**8.936**				

The contact position displacement errors were estimated using simple absolute mean error calculation. However, extensive tests were carried out to retrieve such results. The tests involve the specific ground truth (GT) values for × and Y in a range of −6mm∼+6mm w.r.t force range within 0.1N∼1N. In X-axis, the displacement along the X-axis is incremented by step 1mm while keeping Y value 0mm, and vice versa in the context of Y displacement estimation. The neural network’s performance in terms of estimation of contact position displacement along linear × and Y axes is recorded and reported in Table A2 and Table A3.

**Table A2:** Contact position estimation w.r.t linear × displacement under force ranges (0.1 N∼1 N).

X*_orig_* = −6 mm			X*_orig_* = −5 mm			X*_orig_* = −4 mm			X*_orig_* = −3 mm		
**F** **(N)**	**X*_est_*** **[mm]**	**Error** **[mm]**	**F** **(N)**	**X*_est_*** **[mm]**	**Error** **[mm]**	**F** **(N)**	**X*_est_*** **[mm]**	**Error** **[mm]**	**F** **(N)**	**X*_est_*** **[mm]**	**Error** **[mm]**
0.12	−1.828	4.171	0.13	−2.036	2.963	0.12	−1.530	2.469	0.13	−1.094	1.9056
0.21	−2.875	3.124	0.21	−2.593	2.407	0.22	−2.065	1.934	0.22	−1.588	1.411
0.31	−3.388	2.611	0.31	−2.986	2.013	0.33	−2.435	1.564	0.31	−1.828	1.171
0.40	−3.884	2.115	0.40	−3.352	1.647	0.42	−2.566	1.433	0.40	−1.767	1.232
0.48	−3.997	2.002	0.49	−3.549	1.451	0.50	−2.801	1.198	0.49	−1.798	1.201
0.57	−4.260	1.740	0.59	−3.742	1.257	0.60	−2.817	1.182	0.59	−1.903	1.096
0.66	−4.943	1.056	0.68	−3.643	1.357	0.69	−2.857	1.142	0.69	−1.900	1.099
0.75	−5.610	0.389	0.76	−4.070	0.929	0.77	−3.074	0.926	0.78	−2.115	0.884
0.85	−5.738	0.261	0.85	−4.576	0.423	0.86	−3.288	0.716	0.87	−2.491	0.508
0.95	−5.747	0.252	0.95	−4.818	0.818	0.97	−3.567	0.433	0.96	−2.512	0.487
**X*_orig_* = −2 mm**			**X*_orig_* = −1 mm**			**X*_orig_* = 1 mm**			**X*_orig_* = 2 mm**		
**F** **(N)**	**X*_est_*** **[mm]**	**Error** **[mm]**	**F** **(N)**	**X*_est_*** **[mm]**	**Error** **[mm]**	**F** **(N)**	**X*_est_*** **[mm]**	**Error** **[mm]**	**F** **(N)**	**X*_est_*** **[mm]**	**Error** **[mm]**
0.12	−1.088	0.911	0.14	−1.095	0.095	0.13	−1.026	2.026	0.12	−0.490	2.490
0.22	−1.478	0.521	0.22	−0.949	0.050	0.21	−0.952	1.952	0.22	−0.208	2.208
0.33	−1.409	0.591	0.33	−1.018	0.018	0.33	−0.764	1.764	0.33	−0.608	2.608
0.41	−1.228	0.771	0.41	0.997	0.002	0.41	−0.817	1.817	0.40	−0.356	2.356
0.50	−1.431	0.568	0.50	−0.903	0.096	0.51	−0.275	1.275	0.50	0.166	1.834
0.59	−1.210	0.789	0.60	−0.590	0.409	0.59	0.288	0.711	0.60	0.959	1.040
0.69	−1.297	0.702	0.69	−0.523	0.476	0.69	0.950	0.049	0.70	2.079	0.079
0.78	−1.255	0.744	0.78	−0.334	0.665	0.78	1.421	0.421	0.78	2.567	0.567
0.88	−1.455	0.544	0.88	−0.238	0.761	0.88	1.529	0.592	0.87	2.567	0.567
0.98	−1.519	0.480	0.97	−0.399	0.601	0.97	1.607	0.607	0.98	2.553	0.553
**X*_orig_* = 3 mm**			**X*_orig_* = 4 mm**			**X*_orig_* = 5 mm**			**X*_orig_* = 6 mm**		
**F** **(N)**	**X*_est_*** **[mm]**	**Error** **[mm]**	**F** **(N)**	**X*_est_*** **[mm]**	**Error** **[mm]**	**F** **(N)**	**X*_est_*** **[mm]**	**Error** **[mm]**	**F** **(N)**	**X*_est_*** **[mm]**	**Error** **[mm]**
0.12	−0.461	3.461	0.11	−0.246	4.246	0.12	0.020	4.979	0.13	0.166	5.833
0.23	−0.279	3.279	0.21	0.125	3.874	0.22	0.633	4.366	0.21	0.692	5.308
0.32	−0.401	3.401	0.31	0.958	3.042	0.32	1.588	3.411	0.33	2.673	3.326
0.41	0.093	2.906	0.39	0.983	3.874	0.40	2.317	2.652	0.40	3.060	2.939
0.50	0.942	2.0571	0.50	1.681	2.318	0.50	2.931	2.068	0.49	3.990	2.009
0.60	1.571	1.428	0.59	2.570	1.429	0.58	3.744	1.256	0.57	4.933	1.066
0.69	3.057	0.075	0.69	4.133	0.133	0.67	4.917	0.082	0.67	5.453	0.546
0.78	3.649	0.649	0.77	4.709	0.709	0.76	5.745	0.745	0.75	6.600	0.600
0.88	3.790	0.790	0.87	4.807	0.807	0.86	5.787	0.787	0.84	6.639	0.639
0.97	3.670	0.679	0.97	4.800	0.800	0.96	5.884	0.884	0.94	6.896	0.896

**Table A3:** Contact position estimation w.r.t Y displacement under force ranges (0.1 N∼1 N).

Y*_orig_* = −6 mm			Y*_orig_* = −5 mm			Y*_orig_* = −4 mm			Y*_orig_* = −3 mm		
**F** **(N)**	**Y*_est_*** **[mm]**	**Error** **[mm]**	**F** **(N)**	**Y*_est_*** **[mm]**	**Error** **[mm]**	**F** **(N)**	**Y*_est_*** **[mm]**	**Error** **[mm]**	**F** **(N)**	**Y*_est_*** **[mm]**	**Error** **[mm]**
0.12	0.021	6.021	0.12	0.457	5.457	0.13	−0.345	3.654	0.13	−0.367	2.632
0.20	−0.631	5.3681	0.23	−1.619	3.380	0.21	−0.466	3.533	0.21	−0.781	2.218
0.32	−3.050	2.949	0.32	−2.425	2.574	0.32	−1.793	2.206	0.32	−1.498	1.501
0.40	−4.548	1.452	0.41	−3.545	1.454	0.41	−2.652	1.347	0.41	−1.503	1.496
0.49	−5.476	0.523	0.50	−4.028	0.971	0.51	−3.037	0.962	0.50	−1.936	1.063
0.56	−5.925	0.074	0.58	−4.429	0.570	0.59	−3.173	0.826	0.60	−2.190	0.809
0.66	−5.698	0.301	0.67	−4.453	0.546	0.69	−3.565	0.434	0.69	−2.523	0.476
0.75	−5.811	0.188	0.77	−4.704	0.296	0.76	−3.847	0.152	0.78	−2.960	0.040
0.85	−6.022	0.022	0.85	−4.419	0.419	0.87	−4.485	0.458	0.86	−3.435	0.435
0.95	−6154	0.154	0.95	−4.479	0.479	0.96	−4.464	0.464	0.97	−3.352	0.352
**Y*_orig_* = −2 mm**			**Y*_orig_* = −1 mm**			**Y*_orig_* = 1 mm**			**Y*_orig_* = 2 mm**		
**F** **(N)**	**Y*_est_*** **[mm]**	**Error** **[mm]**	**F** **(N)**	**Y*_est_*** **[mm]**	**Error** **[mm]**	**F** **(N)**	**Y*_est_*** **[mm]**	**Error** **[mm]**	**F** **(N)**	**Y*_est_*** **[mm]**	**Error** **[mm]**
0.13	−0.194	1.805	0.12	0.271	1.271	0.13	0.693	0.306	0.12	1.029	0.970
0.22	−0.718	1.281	0.22	0.268	1.268	0.21	1.234	0.234	0.22	1.736	0.263
0.31	−0.817	1.182	0.32	−0.588	0.411	0.33	0.622	0.377	0.33	1.124	0.875
0.41	−1.444	0.555	0.42	−0.639	0.360	0.41	0.644	0.355	0.41	1.426	0.573
0.50	−1.483	0.516	0.50	−0.929	0.070	0.51	0.757	0.242	0.50	1.585	0.414
0.60	−1.742	0.257	0.60	−0.973	0.026	0.58	0.534	0.465	0.58	1.480	0.519
0.70	−1.807	0.192	0.70	−1.247	0.247	0.70	0.424	0.575	0.69	1.546	0.453
0.78	−2.083	0.083	0.78	−1.283	0.283	0.79	0.691	0.308	0.77	1.576	0.423
0.87	−2.226	0.226	0.87	−1.155	0.155	0.88	0.709	0.290	0.88	1.822	0.177
0.98	−2.271	0.271	0.98	−1.156	0.156	0.98	0.555	0.444	0.98	1.754	0.245
**Y*_orig_* = 3 mm**			**Y*_orig_* = 4 mm**			**Y*_orig_* = 5 mm**			**Y*_orig_* = 6 mm**		
**F** **(N)**	**Y*_orig_*** **[mm]**	**Error** **[mm]**	**F** **(N)**	**Y*_orig_*** **[mm]**	**Error** **[mm]**	**F** **(N)**	**Y*_orig_*** **[mm]**	**Error** **[mm]**	**F** **(N)**	**Y*_orig_*** **[mm]**	**Error** **[mm]**
0.11	1.375	1.624	0.11	1.920	2.079	0.14	2.799	2.200	0.11	2.409	3.590
0.22	1.897	1.102	0.21	2.390	1.609	0.22	3.072	1.927	0.22	3.681	2.318
0.33	2.241	0.758	0.31	0.958	1.484	0.33	3.273	1.726	0.32	3.838	2.162
0.41	2.169	0.831	0.39	2.515	1.031	0.42	3.479	1.520	0.40	4.237	1.762
0.50	2.300	0.699	0.50	2.968	1.077	0.50	3.654	1.345	0.49	4.354	1.645
0.59	2.378	0.621	0.59	2.922	0.846	0.59	3.923	1.076	0.58	4.748	1.241
0.69	2.441	0.558	0.69	3.153	0.775	0.68	4.215	0.784	0.68	5.106	0.893
0.78	2.470	0.529	0.77	3.244	0.581	0.76	4.414	0.585	0.76	5.188	0.811
0.88	2.698	0.302	0.87	3.596	0.403	0.86	4.363	0.363	0.85	5.239	0.760
0.97	2.771	0.228	0.97	3.692	0.307	0.96	4.622	0.377	0.96	5.358	0.642

The contact position estimation errors mentioned in the above two tables are nearly 4 mm∼6 mm in few cases. The main reason behind this is the sensitivity of the tactile sensor setup w.r.t the workbench. It is sightly dependent on the vibrations in the vicinity of the sensor. For instance, when there is a certain activity, such as walking or jumping, happening around the sensor setup, and if the applied force is slightly in the lower magnitudes of 0.1 N, there seems to be vibrations induced into the system. This eventually causes the errors around 4 mm∼6 mm in terms of contact position estimates. However, the overall average error of the contact position still appears to be less than 1.4 mm in × and less than 1 mm in Y, as discussed earlier in Table 5. In addition, the stability of the installation setup heavily influences the performance due to the rotational motions. Therefore, a constructive ground-truth of 45${}^{\circ }$ is calibrated so as to prevent the system installation issues, as shown in Figure A1a. Figure A1 gives a glimpse of various aspects, such as dimensions, camera, and use-cases, that might interest few developers.

## References

[1] Umbaugh S.E. (2010). Digital Image Processing and Analysis: Human and Computer Vision Applications with CVIPtools.

[2] Kakani V., Nguyen V.H., Kumar B.P., Kim H., Pasupuleti V.R. (2020). A critical review on computer vision and artificial intelligence in food industry. J. Agric. Food Res..

[3] Kakani V., Kim H., Basivi P.K., Pasupuleti V.R. (2020). Surface Thermo-Dynamic Characterization of Poly (Vinylidene Chloride-Co-Acrylonitrile)(P (VDC-co-AN)) Using Inverse-Gas Chromatography and Investigation of Visual Traits Using Computer Vision Image Processing Algorithms. Polymers.

[4] Shimonomura K. (2019). Tactile image sensors employing camera: A review. Sensors.

[5] Kakani V., Kim H., Lee J., Ryu C., Kumbham M. (2020). Automatic Distortion Rectification of Wide-Angle Images Using Outlier Refinement for Streamlining Vision Tasks. Sensors.

[6] Kakani V., Kim H., Kumbham M., Park D., Jin C.B., Nguyen V.H. (2019). Feasible Self-Calibration of Larger Field-of-View (FOV) Camera Sensors for the Advanced Driver-Assistance System (ADAS). Sensors.

[7] Luo S., Bimbo J., Dahiya R., Liu H. (2017). Robotic tactile perception of object properties: A review. Mechatronics.

[8] Li W., Konstantinova J., Noh Y., Alomainy A., Althoefer K. (2018). Camera-based force and tactile sensor. Proceedings of the Annual Conference Towards Autonomous Robotic Systems.

[9] Sferrazza C., D’Andrea R. (2019). Design, motivation and evaluation of a full-resolution optical tactile sensor. Sensors.

[10] Yuan W., Mo Y., Wang S., Adelson E.H. Active clothing material perception using tactile sensing and deep learning. Proceedings of the 2018 IEEE International Conference on Robotics and Automation (ICRA).

[11] Yuan W., Li R., Srinivasan M.A., Adelson E.H. Measurement of shear and slip with a GelSight tactile sensor. Proceedings of the 2015 IEEE International Conference on Robotics and Automation (ICRA).

[12] Fearing R.S. (1990). Tactile sensing mechanisms. Int. J. Robot. Res..

[13] Chitta S., Sturm J., Piccoli M., Burgard W. (2011). Tactile sensing for mobile manipulation. IEEE Trans. Robot..

[14] Simonyan K., Zisserman A. (2014). Very deep convolutional networks for large-scale image recognition. arXiv.

[15] Yamaguchi A., Atkeson C.G. (2019). Recent progress in tactile sensing and sensors for robotic manipulation: Can we turn tactile sensing into vision?. Adv. Robot..

[16] Hosoda K., Tada Y., Asada M. Internal representation of slip for a soft finger with vision and tactile sensors. Proceedings of the IEEE/RSJ International Conference on Intelligent Robots and Systems.

[17] Kolker A., Jokesch M., Thomas U. An optical tactile sensor for measuring force values and directions for several soft and rigid contacts. Proceedings of the ISR 2016: 47st International Symposium on Robotics, VDE.

[18] James J.W., Pestell N., Lepora N.F. (2018). Slip detection with a biomimetic tactile sensor. IEEE Robot. Autom. Lett..

[19] Johnsson M., Balkenius C. (2007). Neural network models of haptic shape perception. Robot. Auton. Syst..

[20] Naeini F.B., AlAli A.M., Al-Husari R., Rigi A., Al-Sharman M.K., Makris D., Zweiri Y. (2019). A novel dynamic-vision-based approach for tactile sensing applications. IEEE Trans. Instrum. Meas..

[21] Ma D., Donlon E., Dong S., Rodriguez A. Dense tactile force estimation using GelSlim and inverse FEM. Proceedings of the 2019 International Conference on Robotics and Automation (ICRA).

[22] Wilson A., Wang S., Romero B., Adelson E. (2020). Design of a Fully Actuated Robotic Hand With Multiple Gelsight Tactile Sensors. arXiv.

[23] Taunyazov T., Sng W., See H.H., Lim B., Kuan J., Ansari A.F., Tee B.C., Soh H. (2020). Event-driven visual-tactile sensing and learning for robots. Perception.

[24] Pezzementi Z., Plaku E., Reyda C., Hager G.D. (2011). Tactile-object recognition from appearance information. IEEE Trans. Robot..

[25] Zhang Y., Yuan W., Kan Z., Wang M.Y. Towards Learning to Detect and Predict Contact Events on Vision-based Tactile Sensors. Proceedings of the Conference on Robot Learning.

[26] Begej S. (1988). Planar and finger-shaped optical tactile sensors for robotic applications. IEEE J. Robot. Autom..

[27] Lepora N.F., Ward-Cherrier B. Superresolution with an optical tactile sensor. Proceedings of the 2015 IEEE/RSJ International Conference on Intelligent Robots and Systems (IROS).

[28] Ito Y., Kim Y., Obinata G. (2011). Robust slippage degree estimation based on reference update of vision-based tactile sensor. IEEE Sens. J..

[29] Yang X.D., Grossman T., Wigdor D., Fitzmaurice G. Magic finger: Always-available input through finger instrumentation. Proceedings of the 25th Annual ACM Symposium on User Interface Software and Technology.

[30] Corradi T., Hall P., Iravani P. (2017). Object recognition combining vision and touch. Robot. Biomim..

[31] Luo S., Mou W., Althoefer K., Liu H. (2019). iCLAP: Shape recognition by combining proprioception and touch sensing. Auton. Robot..

[32] Piacenza P., Dang W., Hannigan E., Espinal J., Hussain I., Kymissis I., Ciocarlie M. Accurate contact localization and indentation depth prediction with an optics-based tactile sensor. Proceedings of the 2017 IEEE International Conference on Robotics and Automation (ICRA).

[33] Johnson M.K., Adelson E.H. Retrographic sensing for the measurement of surface texture and shape. Proceedings of the 2009 IEEE Conference on Computer Vision and Pattern Recognition.

[34] Johnson M.K., Cole F., Raj A., Adelson E.H. (2011). Microgeometry capture using an elastomeric sensor. ACM Trans. Graph. (TOG).

[35] Yuan W., Srinivasan M.A., Adelson E.H. Estimating object hardness with a gelsight touch sensor. Proceedings of the 2016 IEEE/RSJ International Conference on Intelligent Robots and Systems (IROS).

[36] Kroemer O., Lampert C.H., Peters J. (2011). Learning dynamic tactile sensing with robust vision-based training. IEEE Trans. Robot..

[37] Meier M., Patzelt F., Haschke R., Ritter H.J. (2016). Tactile convolutional networks for online slip and rotation detection. Proceedings of the International Conference on Artificial Neural Networks.

[38] Chuah M.Y., Kim S. Improved normal and shear tactile force sensor performance via least squares artificial neural network (lsann). Proceedings of the 2016 IEEE International Conference on Robotics and Automation (ICRA).

[39] Kaboli M., Feng D., Cheng G. (2018). Active tactile transfer learning for object discrimination in an unstructured environment using multimodal robotic skin. Int. J. Humanoid Robot..

[40] Gandarias J.M., Garcia-Cerezo A.J., Gomez-de Gabriel J.M. (2019). CNN-based methods for object recognition with high-resolution tactile sensors. IEEE Sens. J..

[41] Sferrazza C., D’Andrea R. (2018). Transfer learning for vision-based tactile sensing. arXiv.

[42] Sato K., Kamiyama K., Kawakami N., Tachi S. (2009). Finger-shaped gelforce: Sensor for measuring surface traction fields for robotic hand. IEEE Trans. Haptics.

[43] Sferrazza C., Wahlsten A., Trueeb C., D’Andrea R. (2019). Ground truth force distribution for learning-based tactile sensing: A finite element approach. IEEE Access.

[44] Qi H., Joyce K., Boyce M. (2003). Durometer hardness and the stress-strain behavior of elastomeric materials. Rubber Chem. Technol..

[45] Moeslund T.B. (2012). BLOB analysis. Introduction to Video and Image Processing.

